# Is Pedometer-Determined Physical Activity Decreasing in Czech Adults? Findings from 2008 to 2013

**DOI:** 10.3390/ijerph13101040

**Published:** 2016-10-24

**Authors:** Jana Pelclová, Karel Frömel, Emil Řepka, Ladislav Bláha, Aleš Suchomel, Igor Fojtík, Dana Feltlová, Petr Valach, Svatopluk Horák, Jiří Nykodým, Michal Vorlíček

**Affiliations:** 1 Faculty of Physical Culture, Palacký University Olomouc, Olomouc 779 00, Czech Republic; karel.fromel@upol.cz (K.F.); svatopluk.horak@upol.cz (S.H.); michal.vorlicek@upol.cz (M.V.); 2 Faculty of Education, University of South Bohemia in České Budějovice, České Budějovice 370 01, Czech Republic; repka@pf.jcu.cz; 3 Faculty of Education, Jan Evangelista Purkyně University in Ústí nad Labem, Ústí nad Labem 400 01, Czech Republic; ladislav.blaha@ujep.cz; 4 Faculty of Science, Humanities and Education, Technical University of Liberec, Liberec 460 01, Czech Republic; ales.suchomel@tul.cz; 5 Pedagogical Faculty, University of Ostrava, Ostrava 702 00, Czech Republic; igor.fojtik@centrum.cz; 6 Faculty of Education, University of Hradec Králové, Hradec Králové 500 03, Czech Republic; dana.feltlova@uhk.cz; 7 Faculty of Education, University of West Bohemia, Plzeň 306 14, Czech Republic; pvalach@ktv.zcu.cz; 8 Faculty of Sports Studies, Masaryk University, Brno 625 00, Czech Republic; nykodym@fsps.muni.cz

**Keywords:** time trends, step-based categories, very active lifestyle, inactive lifestyle, step counts

## Abstract

Objective measured trend data are important for public health practice. However, these data are rare for an adult population. Therefore, the aim of this study was to describe time trends in pedometer-determined physical activity of Czech adults (25–65 years) from 2008 to 2013. Participants were Czech national citizens whose physical activity was assessed objectively using a Yamax Digiwalker SW-700 pedometer (Yamax Corporation, Tokyo, Japan) for seven consecutive days in the period 2008 to 2013. The final sample was 4647 Czech adults [M age 41.4 ± 10 years; M body mass index (BMI) 25.1 ± 3.7 kg/m^2^]. The results showed that men took more steps/day (M (Mean) = 10,014; 95% CI (Confidence Interval) = 9864–10,164) than women (M = 9448; 95% CI = 9322–9673) in all age and BMI groups. Mean steps/day declined from 2008 to 2013 by 852 steps/day in men and 1491 steps/day in women. In the whole sample, the proportion of participants who had a sedentary lifestyle (<5000 steps/day) increased by 5.8%; the proportion taking ≥10,000 steps/day decreased by 15.8%. In 2013, men and women were 2.67 and 2.05 times, respectively, more likely to have a physically inactive lifestyle (<7500 steps/day) than in 2008. Conversely, in 2008, men and women were 1.68 and 2.46 times, respectively, less likely to have very active lifestyle (>12,500 steps/day). In conclusion, this study suggests that there has been a substantial reduction in physical activity in Czech adults over time.

## 1. Introduction

Physical inactivity has major health effects worldwide [1]. It not only causes morbidity and mortality but is also a major economic burden [2]. Therefore, efforts to continue and improve physical activity surveillance, a capacity for intervention, and policy implementation are needed around the world [3].

On a population level, specific quantitative indices are needed for surveillance purposes. Tudor-Locke, Hatano, Pangrazi, and Kang [4] advocated a “zone” approach to assessing the pedometer-determined physical activities of healthy adults: (1) <5000 steps/day (sedentary); (2) 5000–7499 steps/day (low active); (3) 7500–9999 steps/day (somewhat active); (4) 10,000–12,499 steps/day (active); and (5) ≥12,500 steps (highly active). In congruence with the attainment of public recommendations of time spent in moderate-to-vigorous physical activity, the physically active lifestyle threshold is set at 7500 steps/day and physical inactivity is considered to refer to the spectrum of behavior below the moderate-to-vigorous physical activity recommendation covering low active and sedentary zones of steps/day [5]. Healthy adults typically achieve between 4000–18,000 steps/day; hence, 10,000 steps/day seems to be a reasonable and healthy level for this population [6]. On the other hand, the threshold of 12,500 steps is achievable for the substantial proportion of adults in some countries [7,8] and might be associated with health improvement [9].

Trend data provide basic information for different processes in public health practice. Although the potential for information bias from self-reported physical activity is a well-known limitation [10], a large number of trend studies were conducted using self-report instruments. These studies have suggested that the decline in overall physical activity and the increased risk of sedentary time and obesity over the past few decades might be caused mainly by the reduction in occupational physical activity [11,12,13]. Conversely, leisure-time activity levels tend to be increasing over time [12], particularly noticeable in the middle-aged and elderly [14,15]. Data for time trends in transport-related physical activity are less consistent. Long-term decline of transportation activity was observed in the United States [13], but men and women from Canada became more active in transport between 1994 and 2005 [11]. Moreover, a Brazilian population increased their level of transportation physical activity between 2006 and 2009 [16].

Recently published global studies confirmed that there are only a few studies from a small number of high-income countries examining time trends in physical activity by objective methods [3,17]. Pedometers are the most widely used tool in large-scale surveillance studies, as they are inexpensive, easy to use, and generally provide comparable estimates over time [18]. To our knowledge, there are two studies in the adult population using pedometers to establish time trends for physical activity. In a Danish representative survey of adults, a significant decline in steps/day was found between 2007 and 2008 and from 2011 to 2012 [19]. Similarly, in Japan, time trends displayed a decline of mean steps/day from 2000 to 2007 [20]. Whereas the proportion of adults achieving 10,000 steps/day (classified as active) decreased in both countries, increases in the proportion of adults classified as sedentary (taking <4000 steps/day in Japan; taking <5000 steps/day in Denmark) were observed only in Japan. The changes in physical activity were more noticeable in women than in men in both studies.

The main aim of the present study was to describe time trends in the pedometer-determined physical activity of Czech adults from 2008 to 2013. Further aims were to describe time trends in the proportion of Czech adults having (a) an inactive lifestyle and (b) a very active lifestyle.

## 2. Materials and Methods

### 2.1. Study Sample

Participants were recruited for a Czech national 6-year cross-sectional survey called “Physical Activity and Inactivity of the Inhabitants of the Czech Republic in the Context of Behavioral Changes” supported by the Ministry of Education, Youth and Sports of the Czech Republic (No. MSM 6198959221). Standard procedures for identifying households and participants were employed (e.g., every nth house or block of flats and floor were selected and residents with the most recent birthday were recruited) for in-person face-to-face recruitment. For identifying sampling locations in different regions of the Czech Republic, a systematic random sampling method was used in a geocoded national address database. This type of sample selection within the Czech Republic has been used previously [21,22]. We excluded individuals who were not Czech citizens, were disabled, or who lived in the capital of Prague. The city of Prague is quite specific with regard to the number of people living there (over 1 million), workday regime, and leisure time activities. Generally, the living conditions in Prague differ from the rest of the republic as far as the socioeconomic aspects are concerned. The survey was conducted according to the design and methods approved by the Faculty of Physical Culture Ethics Committee at the Palacký University in Olomouc (approval code: 3/2015). Participation was voluntary without any incentives, and each participant signed an informed consent. Participants were allowed to quit the monitoring at any time, and the eventual loss or damage of a monitoring apparatus was not reimbursed by participants.

### 2.2. Physical Activity Assessment

Physical activity was assessed objectively by using a Yamax Digiwalker SW-700 pedometer (Yamax Corporation, Tokyo, Japan). The data were collected in spring and autumn, that is, seasons with comparable weather conditions in the Czech Republic. Participants were instructed to wear the pedometers on the right-hand side of the body in the midline of the right knee during all waking hours, except for during water activities for at least 10 h/day for 7 consecutive days. Furthermore, daily activity logs with compliance instructions were used for subjects to self-record activity patterns so that we could obtain a more accurate picture of the individual’s physical activity (PA) profile and could make some judgments while assessing the data [23].

### 2.3. Procedures and Participants

The initial 11,006 Czech participants, aged 15 to 87 years, were enrolled, out of which 6359 had to be excluded due to incomplete 7-day record sheets (*n* = 1254) and not belonging to the selected age group (25–65 years) (*n* = 4497). The lower limit of sample age (25 years) was set deliberately in order to exclude secondary and university students (different lifestyle habits) to prevent the data distortion. Furthermore, using statistical software, we randomly excluded some participants (*n* = 608) to follow the population distribution provided by the Czech Statistical Office. Hence, the final sample was 4647 Czech adults. The steps/day values lower than 1000 and higher than 25,000 were considered as outliers, and these data were cleaned cautiously with regard to multiple-day monitoring [23]. 

### 2.4. Statistical Analysis

Statistical analysis was undertaken using SPSS 22.0 software (SPSS for Windows, SPSS, Chicago, IL, USA). Means, standard deviations, and proportions were calculated for pedometer-determined physical activity (steps/day, step-defined activity levels according to Tudor-Locke and Bassett) [24]. Descriptive analyses using chi-square test, ANOVA test, and MANOVA test were used to compute any significant gender differences, differences in age, body mass index (BMI), and survey period groups and interaction effects. Ordinal regression (PLUM) was used to calculate the odds ratios of the highest step-based category (very active lifestyle) versus the other step-defined categories according to Tudor-Locke and Bassett [24] separately for men and women. Independent variables in ordinal regression models were time trends (2008–2013), age (25–34 years, 35–44 years, 45–54 years, 55–65 years), and BMI (normal 18.5–24.9 kg/m^2^, overweight 25–29.9 kg/m^2^, obese ≥30 kg/m^2^), with the latter group used as a referent group. Logistic regression models were used for binary outcome (having physically inactive lifestyle) separately for men and women. The upper threshold for a physically inactive lifestyle was set at 7499 steps/day according Tudor-Locke, Craig, Thyfault, and Spence [5]. The independent variables entered into the logistic regression models were similar to ordinal regression. The first group in each category was the referent group in each binary logistic regression analysis.

## 3. Results

### 3.1. Study Population

Characteristics of the study samples in the period 2008 to 2013 are shown in Table 1. No significant differences were found in gender distribution within the six-year survey period. There were 44.3% men (*n* = 2058) and 55.7% women (*n* = 2589) in the whole sample. Mean age of the entire sample was 41.4 (Standard Deviation (SD) = 10.0) years and varied from 39.8 years in 2013 to 42.0 years in 2008. No significant differences were found between survey periods for BMI characteristics. Mean BMI of the six-year sample was 25.1 kg/m^2^ (SD = 3.7) and ranged between 24.7 kg/m^2^ in 2008 and 25.2 kg/m^2^ in 2011 and 2012.

### 3.2. Pedometer-Determined Physical Activity

Steps/day values in relation to survey periods, age, and BMI in men, women, and all participants are shown in Table 2. In Czech adults, MANOVA test shows significant gender (F = 23.05; *p* < 0.001), survey period (F = 16.37; *p* < 0.001), age (F = 18.12; *p* < 0.001), and BMI (F = 38.16; *p* < 0.001) differences in steps/day. However, the interaction effects between factors were not confirmed. Overall, Czech men took more (M = 10,014; 95% CI = 9864–10,164) steps/day than did Czech women (M = 9448; 95% CI = 9322–9673). Men averaged more steps/day than women in all age groups. Age-related declines in mean steps/day were observed in men and women. However, the step/day difference was not statistically significant between the 35–44 and 45–54 years age groups. The steps/day difference between the youngest and the oldest age group was 1143 in men and 1748 in women. According to logistic and ordinal regression models (Table 3 and Table 4), the oldest men and women (55–65 years) were 2.23 times and 2.38 times, respectively, more likely to have a physically inactive lifestyle than the youngest men and women (25–34 years). Conversely, the youngest men and women were 1.93 times and 2.16 times, respectively, more likely to have very active lifestyles.

Inverse linear trends were observed between steps/day and BMI values. The steps/day differences between normal BMI and obese individuals were 1086 in men and 1724 in women. According to logistic and ordinal regression models (Table 3 and Table 4), obese men and women were 1.88 times and 2.43 times, respectively, more likely to have a physically inactive lifestyle than men and women with normal BMI. Conversely, men and women with normal BMI were 1.58 times and 2.34 times, respectively, more likely to meet very active lifestyle steps/day values than obese men and women.

### 3.3. Time Trends in Pedometer-Determined Physical Activity

The time trends for mean steps/day, proportion taking <7500 steps/day (inactive lifestyle), and proportion taking >12,500 steps/day (very active lifestyle) are presented in Table 2, Table 3 and Table 4, respectively. Mean steps/day declined from the highest values in 2008 to 2013 by 852 steps per day in men and 1491 steps per day in women, with the only exception in 2012. In Czech adults between 2008 and 2013, the proportion having a sedentary lifestyle (<5000 steps/day) increased by 5.8%, the proportion taking 5000–7499 steps/day increased by 7.8%, and the proportion taking ≥10,000 steps/day decreased by 15.8%. The proportion having an inactive lifestyle increased by 15.5% in men and 12.3% in women, with noticeable increases in 2010 in both genders. Conversely, the proportion having a very active lifestyle declined by 4.7% in men and 11.7% in women between 2008 and 2013, with a noticeable drop in 2010. 

**Figure 1:**
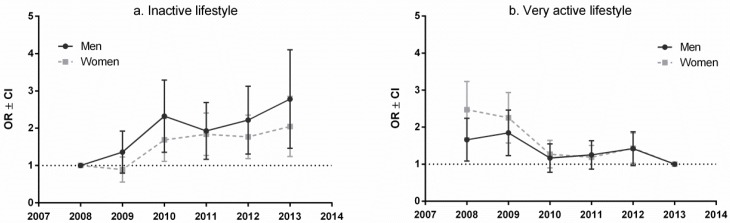
Odds ratios (OR) and confidence interval (CI) for inactive (**a**) and very active (**b**) lifestyle in survey periods 2008–2013.

Figure 1 shows explanatory time trends for increasing inactive lifestyle and decreasing very active lifestyle. In 2013, men and women were 2.67 and 2.05 times, respectively, more likely to have a physically inactive lifestyle than in 2008. Conversely, in 2008, men and women were 1.68 and 2.46 times, respectively, less likely to have very active lifestyle.

## 4. Discussion

In the Czech Republic, the trend studies on physical activity in adults are scarce, with most studies examining physical activity in children and adolescents with self-report tools [25,26]. This is the first study describing time trends in adults’ physical activity objectively using pedometers and presenting data from a Czech national six-year survey. Results from this study could be used to inform policy and practice in physical activity-related areas not only in the Czech Republic but also in other Central and Eastern European countries that have undergone similar substantial economic transformations during this time. Such transformations can significantly influence the health conditions, lifestyle, and physical activity levels of inhabitants [27,28].

In this study, we aimed to examine time trends in pedometer-determined physical activity. Significant decline in steps/day in this study is consistent with the findings of other studies confirming the substantial reduction in adults’ physical activity over time [19,20]. In line with these studies, a greater decline in steps/day occurred among women (1491 steps/day) than men (852 steps/day), with no interaction effect between survey period and gender, or survey period and age. Decline in physical activity seems to be greater in Czech men and women than in the Japanese study [20], with a steps/day reduction of 529 in men and 857 in women between 2000 and 2007, and in the Danish study [19], which reported a decline in both sexes (446 steps/day) between 2007 and 2012. The decline of physical activity in Czech adults might be associated with the increasing socio-economic statute in the Czech Republic. From the data from the 2001 and 2011 Czech Census of Population and Housing, it is apparent that, for example, the number of automobiles per 1000 inhabitants increased by 26%, and the number of households with internet access increased from 5.8% to 61.7% over 10 years [29].

We found a significantly growing proportion of Czech individuals with an inactive lifestyle and a diminishing proportion of those with a very active lifestyle. These results can explain the steps/day decline of Czech adults between 2008 and 2013. There is noticeable difference between men and women. Whereas the proportion of participants who had an inactive lifestyle increased more in men than in women (difference 3.2%), the proportion having a very active lifestyle declined by 7% more in women than in men. This suggests that the greater decline in steps/day that occurred during the six years among women was reflected in a lower proportion of women with a very active lifestyle rather than in a higher proportion of women with an inactive lifestyle. Conversely, in the Danish study [19], the overall reduction in the PA level was reflected in a higher proportion of especially women having a sedentary or low-active lifestyle. In line with our findings, the proportion of adults having a sedentary lifestyle showed reciprocal change compared with the proportion of those taking ≥10,000 steps/day in the Japanese population [20]. However, the increase in the proportion having a sedentary lifestyle was more pronounced in Japanese women in recent years.

On the basis of the findings from international studies using self-report [30,31] and objective instruments [32,33], the Czech Republic belongs among countries with a high level of overall physical activity, with a high amount of moderate-to-vigorous physical activity largely accumulated through walking. This is also apparent in steps/day values. Czech adults took on average 9765 steps/day, which is more than Japanese [20], Danish [19], Americans [34], and Finns [35], and comparable to Belgians [7], Australians [36], and UK adults [37]. Czech men achieved significantly more steps/day than women, which is in line with studies that reported the gender differences in daily step counts [7,34,36,37]. However, in some studies, no differences in pedometer-determined physical activity between men and women were found [19,20]. In contrast to other studies, in the Finnish study, women took significantly more steps than men [35]. Age-related decline in steps/day was noticeable, especially between middle-aged and older adults, as suggested in other studies [7,20,34]. In this study, a negative association between steps/day and BMI was also observed. Moreover, obese individuals were more likely to be inactive and achieved 1269 fewer steps/day than normal weight individuals. These findings, which are in line with other studies [34,36], show that the unchanged situation over six years might be a challenge for Czech public health initiatives. On the other hand, an increase in approximately 1500 steps/day over individual baseline is an achievable aim of step-based interventions in adults [38].

To our knowledge, this is the first objective study in Central Europe describing the time trends of physical activity in an adult population. Moreover, the most recent data are included. On the basis of previous findings about the existing day-of-the-week variability of step counts per day in the Czech population ≤64 years with Sunday as the least-active day [39], seven days of data collection were chosen to more realistically estimate habitual volume of physical activity. This study has limitations. First, the habitual walking behavior could have been influenced by the use of unsealed pedometers. Whereas some studies reported minimal differences between sealed and unsealed step counts in adult samples [40,41], Clemes and Deans [42] suggested that reactivity to unsealed pedometers with step count recording lasts for a period of one week. Use of self-reported steps/day might lead to overestimation of physical activity, but we can be confident in the evidence for time trends, as the same methods of data collection were used in each year of the six-year survey. Second, the participants in this study were asked to wear a pedometer during waking hours for at least 10 h/day and record the number of steps in a step diary. However, they did not record time of attachment and removal of the pedometer; if we had, that would have allowed us to examine cadence (steps/min) for further consideration of the data. For example, in the Danish trend study, gender difference in steps/day was significant; however, when cadence was considered, differences between genders were not significant [19]. On the other hand, Inoue et al. [20], in a Japanese study, advocated for one-day monitoring without a cadence record for surveillance to assess population level of physical activity. Third, the inhabitants of the capital of Prague were not included into analyses due to different socioeconomic conditions. Thus, the results may not be fully generalizable to the Czech adult population.

## 5. Conclusions

This national survey suggests a decreasing level of physical activity in Czech adults over a six-year period. Moreover, this study indicates that an increasing proportion of adults have an inactive lifestyle, and a decreasing proportion of those have a very active lifestyle. In 2013, 36.9% of Czechs achieved the recommended 10,000 steps/day, and 8.8% of Czech adults were sedentary (taking <5000 steps/day). Hence, considering that almost two-thirds of the Czech adult population is insufficiently active, presumably continuing a downward trend in physical activity in the coming years, this study sends an important message from a public health perspective. However, these findings, suggesting a reduction in physical activity, have to be confirmed by further studies employing a representative sample of Czech adults.

## Figures and Tables

**Table 1:** Characteristics of the study samples in the survey periods 2008–2013.

	2008	2009	2010	2011	2012	2013	p*
Gender [*n* (%)]							
Men	216 (42.8)	331 (48.3)	357 (45.7)	602 (42.7)	393 (43.5)	159 (44.2)	0.204
Women	289 (57.2)	355 (51.7)	424 (54.3)	809 (57.3)	511 (56.5)	201 (55.8)	
Age [years; mean (SD)]	42.0 (11.2)	41.0 (9.5)	41.7 (9.7)	41.6 (10.1)	41.3 (9.8)	39.8 (9.6)	0.020
Age groups [*n* (%)]							
25–34 years	154 (30.5)	192 (28.0)	201 (25.8)	378 (26.8)	245 (27.1)	118 (32.8)	<0.001
35–44 years	137 (27.1)	213 (31.0)	240 (30.7)	444 (31.5)	289 (32.0)	116 (32.2)	
45–54 years	135 (26.7)	240 (35.0)	273 (35.0)	465 (33.0)	299 (33.1)	106 (29.4)	
55–65 years	79 (15.6)	41 (6.0)	67 (8.7)	124 (8.8)	71 (7.9)	20 (5.6)	
BMI [kg/m^2^; mean (SD)]	24.7 (3.5)	25.1 (3.6)	25.0 (3.5)	25.2 (3.8)	25.2 (3.7)	25.0 (3.7)	0.170
BMI groups [*n* (%)]							
Normal BMI	284 (56.2)	361 (52.6)	425 (54.4)	740 (52.4)	477 (52.8)	184 (51.1)	0.350
Overweight	182 (36.0)	255 (37.2)	294 (37.6)	515 (36.5)	334 (36.9)	145 (40.3)	
Obesity	39 (7.7)	70 (10.2)	62 (7.9)	156 (11.1)	93 (10.3)	31 (8.6)	

BMI, body mass index; p*—values represent differences between the periods using chi-square and ANOVA tests.

**Table 2:** Steps/day values in relation to survey periods, age, and body mass index (BMI) in men, women, and all participants.

	Steps/Day								
	All			Men			Women		
	*M*	*SD*	p*	*M*	*SD*	p*	*M*	*SD*	p*
Survey period									
2008	10,497	3293	<0.001	10,543	3395	<0.001	10,463	3320	<0.001
2009	10,389	3404		10,696	3565		10,103	3226	
2010	9417	3267		9696	3482		9182	3059	
2011	9340	3261		9736	3413		9046	3112	
2012	9715	3459		10,031	3520		9472	3396	
2013	9233	3297		9600	3197		8941	3353	
Age (years)									
25–34	10,440	3331	<0.001	10,577	3449	<0.001	10,270	3473	<0.001
35–44	9866	3222		10,242	3734		9659	3384	
45–54	9776	3532		10,008	3672		9598	3411	
55–65	8903	3573		9434	3650		8522	3474	
BMI									
normal	9950	3299	<0.001	10,385	3467	<0.001	9757	3210	<0.001
overweight	9604	3404		9915	3453		9102	3264	
obesity	8681	3276		9267	3427		8044	2983	

p*—values represent differences between the survey periods, age groups, and BMI groups for all, men, and women using ANOVA tests.

**Table 3:** Association between a physically inactive lifestyle (<7500 steps/day) and age, body mass index (BMI), and year of monitoring.

	Physically Inactive Lifestyle (<7500 Steps/Day)			
	Men		Women	
	*n (%)*	*OR (CI)*	*n (%)*	*OR (CI)*
Survey period				
2008	37 (15.2)		69 (21.0)	
2009	57 (17.2)	1.24 (0.77–1.99)	67 (18.9)	0.91 (0.61–1.36)
2010	101 (28.3)	2.34 *** (1.50–3.66)	128 (30.2)	1.74 ** (1.21–2.49)
2011	152 (25.2)	2.00 ** (1.28–2.98)	263 (32.5)	1.91 *** (1.37–2.65)
2012	108 (27.5)	2.24 *** (1.44–3.48)	161 (31.5)	1.83 ** (1.29–2.59)
2013	50 (30.7)	2.67 *** (1.60–4.46)	70 (33.3)	2.05 ** (1.35–3.12)
Age (years)				
25–34	179 (19.9)		133 (21.5)	
35–44	126 (24.6)	1.22 (0.93–1.62)	258 (27.8)	1.35 * (1.05–1.73)
45–54	169 (25.6)	1.27 (0.98–1.65)	264 (30.8)	1.47 ** (1.14–1.90)
55–65	61 (36.3)	2.23 *** (1.53–3.25)	103 (44.0)	2.38 *** (1.69–3.35)
BMI				
normal	157 (19.8)		433 (24.6)	
Overweight	268 (25.2)	1.24 (0.98–1.57)	222 (33.6)	1.42 *** (1.16–1.74)
Obesity	80 (34.0)	1.88 *** (1.34–2.62)	103 (47.7)	2.43 *** (1.80–3.28)

* *p* < 0.05; ** *p* ≤ 0.01; *** *p* ≤ 0.001; OR, odds ratio; CI, confidence interval.

**Table 4:** Odd ratios (ORs) and 95% confidence intervals (CIs) for step-defined very active lifestyle.

	Very Active Lifestyle (>12,500 Steps/Day)			
	Men		Women	
	*n (%)*	*OR (CI)*	*n (%)*	*OR (CI)*
Survey period				
2008	51 (23.6)	1.68 ** (1.16–2.42)	74 (25.6)	2.46 *** (1.78–3.4)
2009	86 (26.0)	1.75 *** (1.25–2.46)	77 (21.7)	2.14 *** (1.57–2.92)
2010	72 (20.2)	1.11 (0.79–1.55)	55 (13.0)	1.21 (0.9–1.64)
2011	117 (19.4)	1.2 (0.88–1.64)	104 (12.9)	1.13 (0.86–1.5)
2012	90 (22.9)	1.35 (0.97–1.88)	90 (17.6)	1.37 * (1.23–1.83)
2013	29 (18.2)		29 (14.4)	
Age (years)				
25–34	185 (25.8)	1.93 *** (1.42–2.62)	126 (22.1)	2.16 *** (1.63–2.87)
35–44	98 (19.1)	1.49 * (1.09–2.04)	130 (14.0)	1.55 *** (1.19–2.02)
45–54	138 (20.9)	1.48 * (1.09–2.01)	141 (16.4)	1.61 *** (1.23–2.09)
55–65	24 (14.3)		32 (13.7)	
BMI				
normal	187 (24.6)	1.58 *** (1.21–2.07)	319 (18.6)	2.34 *** (1.8–3.04)
Overweight	217 (20.4)	1.30 * (1.04–1.73)	96 (14.5)	1.74 *** (1.32–2.3)
Obesity	41 (17.4)		14 (6.5)	

* *p* < 0.05; ** *p* ≤ 0.01; *** *p* ≤ 0.001; OR, odds ratio; CI, confidence interval; BMI, body mass index.
